# Supplementary material for: Relational responsibilities: Researchers perspective on current and progressive assessment criteria: A focus group study
Source: PLoS One. 2024 Sep 4;19(9):e0307814. doi: 10.1371/journal.pone.0307814 (PMC11373834; doi:10.1371/journal.pone.0307814)
Supplement: S1 File — (PDF) [file pone.0307814.s001.pdf]

medisch ethische toetsingscommissie

Van der Boechorststraat 7
postbus 7057
1007 MB Amsterdam

telefoon 020 444 5585
kamer H-565

www.vumc.nl/METc
METc@vumc.nl

J. Tijdink, MD, PhD
Afdeling Epidemiologie & Biostatistiek

Correctie d.d. 11-7-2018

VUmc

onderwerp	ons kenmerk	datum
niet-WMO advies	2018.209	26 april 2018

Geachte heer Tijdink,

Het Dagelijks Bestuur van de Medisch Ethische Toetsingscommissie VU medisch centrum heeft uw onderzoek **Optimizing the Responsible Researcher: Towards fair and constructive academic advancement (ORR)** besproken in de vergadering van 17/04/2018.

Het onderzoek valt niet onder de reikwijdte van de Wet Medisch-wetenschappelijk Onderzoek met mensen (WMO).

Het oordeel is gebaseerd op de volgende documenten:

Sectie	Onderwerp	Versie
A1	aanbiedingsbrief	d.d. 11-4-2018
A1	commentaar METc	d.d. 25-4-18
C1	onderzoeksprotocol	versie 1 d.d. 7-3-2018
E11	informatiebrief	versie 1 d.d. 11-4-2018
E2	toestemmingsverklaring	versie 1 d.d. 11-4-2018

Het Dagelijks Bestuur van de Medisch Ethische Toetsingscommissie VU medisch centrum wijst u erop dat hoewel het ingediende onderzoek niet onder de reikwijdte van de WMO valt, andere wet- en regelgeving (mogelijk) wel van toepassing is, waaronder:

- WGBO (Wet Geneeskundige BehandelingsOvereenkomst);
- WBP (Wet Bescherming Persoonsgegevens), zie www.cbpweb.nl;
- Code Goed Gedrag (Gedragscode gezondheidsonderzoek: gebruik medische gegevens in wetenschappelijk onderzoek), zie www.federa.org;

Zelfstandig bestuursorgaan
De METc VUmc is een erkende onafhankelijke toetsingscommissie en geeft als zelfstandig bestuursorgaan (ZBO) oordelen die landelijk geldig zijn.

202.000.235

- Code Goed Gebruik (Gedragscode Verantwoord omgaan met lichaamsmateriaal ten behoeve van wetenschappelijk onderzoek, 2011), zie www.federa.org;
- Biobanken: Reglement toetsing biobank VUmc, zie https://www.vumc.nl/afdelingen/METc/biobank/;
- WBO (Wet Bevolkings Onderzoek), zie http://www.vumc.nl/afdelingen/METc/wetgeving/wetbevolkingsonderzoek/.

To whom it may concern

We are pleased to confirm that the Medical Research Involving Human Subjects Act (WMO) does not apply to the above mentioned study and that an official approval of this study by our committee is not required.

The Medical Ethics Review Committee of VU University Medical Center is registered with the US Office for Human Research Protections (OHRP) as IRB00002991. The FWA number assigned to VU University Medical Center is FWA00017598.

Met vriendelijke groet,
namens de Medisch Ethische Toetsingscommissie VU medisch centrum,

i.o.

prof. dr. C. Boer, voorzitter

c.c.: Afdelingshoofd Epidemiologie & Biostatistiek (dr. J. Berkhof)
