# Supplementary material for: Relational responsibilities: Researchers perspective on current and progressive assessment criteria: A focus group study
Source: PLoS One. 2024 Sep 4;19(9):e0307814. doi: 10.1371/journal.pone.0307814 (PMC11373834; doi:10.1371/journal.pone.0307814)
Supplement: S1 Table — (DOCX) [file pone.0307814.s002.docx]

**Supplementary Table 1 Translation of the Dutch quotes**

Translation of the quotes that were handled in Dutch and needed translation.

| Research Integrity | |
| --- | --- |
| Dutch Quote | English Translation |
| *‘En integriteit. Ik kan nog niet goed zien hoe je dat zou moeten beoordelen. Wat natuurlijk voor verantwoordelijk wetenschapper wel ook heel belangrijk. Dat zijn een beetje de kwalitatieve maten die je zou moeten uitvragen of zo. Of je zou mensen elkaar moeten laten beoordelen.’* | Senior Researcher UMC 1:  *And integrity: I cannot see yet how one should assess this. It is very important, of course, for a responsible scientist, to have some of these measures that you should ask for in a more qualitative manner or that should be part of the way people assess one another* |
| Focus group in English, no translation needed | Senior Researcher UMC 2:  *The only thing that comes to my mind that would be an unbiased, but really tricky, way to sort of assess honesty, is to look at how someone’s work is followed up and reproduced independently, right, after publication* |
| *‘…want dat is ook verantwoord onderzoek heb je ook misschien wel verantwoording over je onderzoekers’* | Junior researcher UMC 3:  *‘being responsible for your researchers is also part of responsible research’* |
|  | Junior researcher UMC 2:  *‘people who enjoy the game, but that are not doing responsible research, they stick around too much’* |
| *‘…Maar juist als je naar het systeem van nu kijkt, sorteert het enorm voor op een heel specifiek, eng persoonlijkheidstypetje.’* | Junior researcher UMC 1:  *‘When you look at the system as it is right now, it presorts quite significantly on a very specific, scary personality type’* |
| *‘Samenwerken, de samenwerkingscompetentie. Die moet worden beoordeeld….’* | Senior researcher UMC 3 *:*  *‘collaboration, the competence to collaborate, has to be assessed’* |
| *‘Je zou ook een soort van, nou ja, we hebben functionerend een teamprijs, maar je zou ook zoiets van de teamplayerprijs kunnen hebben.’* | senior researcher UMC 3:  *‘You could also have like, well, a prize for a well-functioning team; you could have something like a team player prize’* |
| PhD Supervision theme | |
| *‘nou ja, weet hoe het hoort en laat zien hoe het hoort. En daar waar mensen daarvan denken te kunnen afwijken, mensen ook tot de orde roepen*.’ | Senior researcher UMC 3:  ‘*well, you know how things ought to be and show that too. And you straighten people out when they think they can deviate from it’* |
| *‘En dat betekent dat je niet alleen investeert in wetenschappelijke opleiding, maar ook in de breedte in de ontwikkeling van een persoon.’* | Senior researcher UMC 3 *:*  *‘not only have to invest in scientific training but in someone’s personal development in the wider sense of the term’* |
| *‘En dat leer je op een gegeven moment en dan denk je, oke. Dat je daar wel bewuster mee om gaat. De interactie met je mensen*.’ | Senior researcher UMC 3:  *‘you grow into this at a certain moment’ through the ‘interaction with your people’* |
| *‘Ik denk overigens wel dat als je goed getraind wordt en dat het eerder als het ware aan je uitgelegd wordt’.* | Senior researcher UMC 3:  *‘when you are trained well and when it is, kind of, explained to you’* |
| Teaching | |
| *‘Onderwijs geven of uitdragen naar de maatschappij. Dat dat er ook bij hoort wat nu niet wordt gezien als ook een goede wetenschapper’* | Junior researcher UMC 1 *:*  *‘To teach or to disseminate things to society: that does belong to it but is currently not considered to be a way to be a good scientist’* |
| ‘dat ons onderwijs soms, bij sommige mensen niet hun grootste hobby en dan doen ze dat dan toch en daar proberen ze weer een goede evaluatie op een of andere manier in te fietsen’ | Senior researcher UMC 3 :  ‘For some people, teaching is not their greatest hobby. And then they still do it and try to somehow get a good evaluation out of it’ |
| ‘Je moet onderzoek doen, en het liefst sociaal relevant, en je moet ook nog goed in onderwijs zijn’ | Senior researcher UMC 3 :  *‘You have to conduct research, and create societal relevant work and you have to be good at teaching too’* |
| From Relational responsibilities to relational assessment | |
| *‘Je hebt de functie van een rolmodel in de onderzoeksgroep, dat je ook, nou ja, weet hoe het hoort en laat zien hoe het hoort. En daar waar mensen daarvan denken te kunnen afwijken, mensen ook tot de orde roepen.’* | Senior researcher UMC 2:  *‘In a research group, you function as a role model in that you also, well, know how things should go and show how things should go. And to call people to order in case they think they can deviate from this.’* |
| *‘Maar ik zou verantwoord meer vinden iemand die zich wat dat betreft een beetje dienstbaar opstelt en niet zozeer te veel egoïstisch.’* | Senior researcher UMC 2 *:*  *‘I would think that ‘responsible’ is more applicable to someone who intends to be of service to others and does not act in too selfish a way’* |
| *‘Narcisme is natuurlijk een uitgesproken persoonlijkheid…. dat pas niet bij verantwoordelijke onderzoekers.’* | *Junior Researchers UMC 1:*  *‘narcissism is an outgoing personality, that is not something compatible with responsible researchers* |
|  |  |
| ‘Want het is haast zo dat het ondanks het systeem, moet je een teamplayer blijven. Want het systeem is natuurlijk heel erg afrekenend op individuen.’ | Senior researcher UMC 3*:*  *‘Because it is almost the case that you remain a team player despite the system. Because the system* *holds individuals accountable of course’* |
| *Relational Assessment 1: Reward team effort and team science* | |
| ‘Volgens mij is het grootste struikelblok dat alle criteria zich richten op het individu. Terwijl we, zeker in deze discussie vandaag, willen benadrukken dat wetenschap een team proces is, iets gezamenlijks’ | *Senior Researcher UMC 2:*  *‘According to me, the biggest bottleneck is that these criteria always apply to the individual. While we, in this conversation, emphasize that it is a collective effort, a team process’* |
| *Relational assessment theme 2: provide 360 degrees of feedback* | |
| misschien moet je mensen ook laten interviewen door commissies of ja, iets dergelijks. Dat je veel meer zicht krijgt op die persoon daadwerkelijk en hoe hij opereert in zo’n team. | Senior research UMC 1:  *‘perhaps people should be interviewed by commissions or something like that. That you get a way better picture of the person and how he operates in such a team’* |
